# β-tricalcium phosphate/gelatin composite scaffolds incorporated with gentamycin-loaded chitosan microspheres for periodontal regeneration in class II furcation defects in dogs

**DOI:** 10.1007/s00784-025-06582-0

**Published:** 2025-10-29

**Authors:** Mohamed Hamdy Helal, Ahmed Noaman Ali, Yasmin Hamdy, Malak Yousef Mohamed Shoukheba, Khaled M. Ali, Moustafa Nabil Aboushelib

**Affiliations:** 1 Oral Medicine, Periodontology, Oral Diagnosis, and Radiology Department, Faculty of Dentistry, Tanta University, Tanta, 35511 Egypt; 2 Department of Basic Medical and Dental Sciences, Faculty of Dentistry, Zarqa University, Zarqa, Jordan; 3 Oral Pathology Department, Faculty of Dentistry, Tanta University, Tanta, Egypt; 4 Department of Surgery, Faculty of Veterinary Medicine, Cairo University, Giza, Egypt; 5 Dental Biomaterials Department, Faculty of Dentistry, Alexandria University, Alexandria, Egypt

**Keywords:** Β-tricalcium phosphate/gelatin composite scaffolds incorporated with gentamycin-loaded chitosan microsphere, Periodontal regeneration, Class II furcation defects

## Abstract

**Objectives:**

The present study aimed to evaluate the effect of β-tricalcium phosphate/gelatin composite scaffolds incorporated with gentamycin-loaded chitosan microspheres for periodontal regeneration in Class II Furcation Defects in Dogs.

**Materials and methods:**

24 bilateral class II furcation defects, 5 mm height, and 3 mm depth, were surgically created on the buccal surface of mandibular premolars and allowed to become chronically healed in six mongrel male dogs. Dogs were randomly divided into three groups: Group I received β-tricalcium phosphate/gelatin composite scaffolds incorporated with gentamycin-loaded chitosan microspheres (CMs (GM)-β-TCP/gelatin composite scaffolds). Group II received the same scaffolds without gentamycin. Group III received open flap debridement (OFD) as a control. The animals were sacrificed at 8 weeks post-operatively for histological and histomorphometry analysis (*n* = 8, α = 0.05).

**Results:**

New regenerative tissue was significantly (*P* < .001) greater in both the test groups, as they showed the highest percentage of newly formed bone height, newly formed bone area, newly formed cementum, and newly formed periodontal tissues than the control. At the same time, epithelial down growth was predominant in the control group.

**Conclusion:**

Within the limitations of this study, the novel scaffolds improved periodontal regeneration and wound healing in chronic class II furcation defects.

**Clinical relevance:**

The Bone regeneration property of (CMs (GM)-β-TCP/gelatin composite scaffolds) is implicated in periodontal bone defect treatment and pre-implant alveolar bone regeneration.

**Supplementary Information:**

The online version contains supplementary material available at 10.1007/s00784-025-06582-0.

## Introduction

The bifurcation or trifurcation of multirooted teeth is ultimately affected by attachment loss due to the uncontrolled progression of inflammatory periodontal disease [1]. The complex structure of the furcation area makes it very difficult to clean during regular periodontal treatments. As a result, dealing with furcation involvement in molars is considered one of the most challenging conditions in clinical periodontics [2]. Several therapies have been developed to treat furcation lesions, including non-surgical periodontal therapy (SRP) and surgical procedures such as root resection, hemi-sectioning, root debridement, regenerative therapy, and tissue engineering [3]. The therapeutic strategy is less certain in cases with class II furcation involvement. Although the efficacy of regeneration as a treatment for this kind of injury has been demonstrated, treatment-type predictability is still a significant obstacle. However, during the past few decades, there has been a lot of interest in tissue regeneration and reconstruction therapies [4]. Autogenous, allogeneic, xenograft, and alloplastic bone grafts, among other types of bone grafts [1, 2, 4–6]. However, each has its drawbacks and limitations when it comes to simultaneously completing the morphological and functional repairs of the defects [2, 3].

Artificial scaffolds can be implanted at the site of the bone deficiency to circumvent these issues and stimulate osteogenesis in both quantity and quality [1, 7]. Nowadays, the fundamental approach of bone regeneration involves introducing one or more of the basic components required for bone formation, such as scaffolds, osteoprogenitor cells (mesenchymal stem cells), bioactive factors, or genes into the target site to promote cellular proliferation and differentiation while directing the body’s natural ability to repair tissue [8, 9]. Recent studies have demonstrated that the implantation of an acellular biodegradable scaffold into the target region, with or without a bioactive component, can attract mesenchymal stem cells and/or osteoprogenitor cells from the surrounding area, which can then regenerate the bone [9–11]. The primary strategy to reduce harmful side effects and enhance therapeutic efficacy is the use of biological materials as drug carriers [12, 13]. Microspheres (MSs) are microscale customizable platforms that can improve medicine administration to specific sites and biological dissemination [14]. A typical option for MS manufacturing is chitosan (CS). CS is an inexpensive, naturally occurring biocompatible polysaccharide [15, 16]. Above all, CS possesses pharmacological properties like antibacterial and bactericidal actions [17, 18]. Treatment of infected bone defects has shown promising results with β-tricalcium phosphate/gelatin composite scaffolds infused with gentamycin-loaded chitosan microspheres [19].

The rationale for selecting the scaffold components in this study has been clarified to emphasize their complementary biological and mechanical functions. β-tricalcium phosphate (β-TCP) is widely recognized as an osteoconductive and biodegradable biomaterial that provides a temporary three-dimensional framework for new bone formation and mineral deposition [20, 21]. However, β-TCP alone is brittle and lacks sufficient handling properties, which limit its clinical application in periodontal defects. To overcome this, gelatin, a naturally derived polymer, was incorporated to enhance scaffold biocompatibility and hydrophilicity, while also providing Arg–Gly–Asp (RGD) motifs that promote cell adhesion, proliferation, and tissue integration [22, 23]. Additionally, chitosan microspheres (CMs) were introduced as a localized drug-delivery system. Chitosan is a biodegradable polysaccharide with inherent antibacterial and wound-healing properties, and when engineered into microspheres, it enables controlled release of therapeutic agents [24, 25]. In this study, the microspheres were loaded with gentamicin to provide sustained antimicrobial activity at the defect site, thereby reducing infection risk and creating a favorable environment for periodontal wound healing. The combination of β-TCP, gelatin, and gentamicin-loaded chitosan microspheres was therefore expected to act synergistically by providing mechanical stability and osteoconductivity, enhancing soft- and hard-tissue integration, and maintaining local infection control—factors essential for predictable periodontal regeneration [26, 27]. To the best of our knowledge, no research has been done on how it affects class II furcation periodontal regeneration; hence, the purpose of this study was to assess how it affects canine model periodontal regeneration. The null hypothesis proposed was that, in comparison to open debridement, similar results would be achieved with β-tricalcium phosphate/gelatin composite scaffolds with or without gentamycin-loaded chitosan microspheres.

## Materials and methods

This research was carried out in the Department of Surgery, Anesthesiology, and Radiology, Faculty of Veterinary Medicine at Cairo University. The Study protocol was approved by the ethics committee of Tanta University, specifying conditions and constraints for conducting and publishing studies involving animal models (approval no #R-OMPDR-6–23-3) and followed the ARRIVE guideline [28]. Scaffold Preparation β-tricalcium phosphate/gelatin composite scaffolds incorporated with gentamycin-loaded chitosan microspheres were prepared using the method of Yu Liu et al. [19].

The sample size was calculated using G Power version 3.1.9.2 [29]. The minimal sample size was calculated based on a previous study aimed to evaluate the histologic and histomorphometric effects of hyaluronic acid (HA) gel with or without acellular dermal matrix allograft (ADMA) on periodontal regeneration in Class II furcation defects in dogs [30] suggested that HA with ADMA positively affects periodontal regeneration and wound healing in Class II furcation defects. The sample size was calculated to assess the difference in newly formed bone area (primary outcome) among the three groups. Based on Tella et al. (2023) [30] results, adopting a power of 80% ((β = 0.20) to detect a standardized effect size in the newly formed bone area (primary outcome) of 0.518, and a level of significance 5% (α error accepted = 0.05), the minimum required sample size was found to 8 specimens per group (number of subgroups = 3) (Total sample size = 24 specimens) [29]. Any specimen loss from the study sample due to a processing error was replaced to maintain the sample size [31].

### Scaffold preparation

Chitosan microspheres (CM) loaded with Gentamycin (Gm) were fabricated using the microcapsulation method [32] after stirring and dissolving 10 g of chitosan and 5 g of Gentamycin in 500 mL of 1% acetic acid (Millipore Merck, Germany) solution. 1.8 g of CMs (GM) and 1.1 g of Nano β-TCP (less than 100 nm, spheroidal shape, Nano Gate Company, Egypt) were added to 5 mL of 18% gelatin (Loba–Chemie, India) solution at 37˚C. 150 mL of tripolyphosphate (TPP) (0.2% w/v) was added in the dropwise method to precipitate the loaded microspheres. Final precipitation was assisted using a sodium hydroxide solution. After constant stirring at 5000 rpm for 5 min, 1.5 mL of 1% genipin solution was added and stirred for another 30 min. The mixture was poured into prefabricated casts of the defect size and freeze-dried overnight [19]. The casts allowed the fabrication of scaffolds that precisely fit the defect size.

Scanning electron microscopy (SEM) was used to analyze the composite scaffold’s shape and structure. In brief, the composite scaffold specimens were secured to the platform after being cut into thin parts and secured firmly onto the platform. Following platinum-palladium sputter coating, the sections were analyzed using a Field Emission Scanning Electron Microscope (FESEM, Quattro S, Thermo Scientific). Mercury porosimetry was used to evaluate the average pore size and distribution. Radiograph diffraction analysis (RDX) was used to determine the elemental surface composition.

### Experimental animals and their housing protocol

All specimens were sterilized using gamma radiation (the Egyptian Atomic Energy Authority). Six mongrel dogs, weighing 12 to 14 kg, were chosen for the study and were kept at an animal healthcare facility in the Faculty of Veterinary Medicine at Cairo University. The dogs were in good general health. They were provided with a balanced diet of milk, broth, and meat during the study period. All animals were kept in individual stainless-steel cages with direct access to water, proper ventilation, as well as 12-hour light/dark cycles. Twenty-four mandibular premolar teeth of six mongrel male dogs (aged two years) were used. The dogs were given a subcutaneous injection of atropine (0.05 mg/kg; Kwang Myung Pharmaceutical) to induce anesthesia. After 10 min of premedication, anesthesia was induced by injecting a mixture of 2 mg/kg Xylazine (Xyla-Ject; Adwia Pharmaceuticals) and 5.5 mg/kg ketamine hydrochloride (KETAMAX-50; Troikaa Pharma) into the cephalic vein of the forelimb and was maintained with inhalation anesthesia. As well as a local infiltration (2% with 1:100,000 epinephrine, Art pharms, Egypt).

### Surgical protocol

**Fig. 1:**
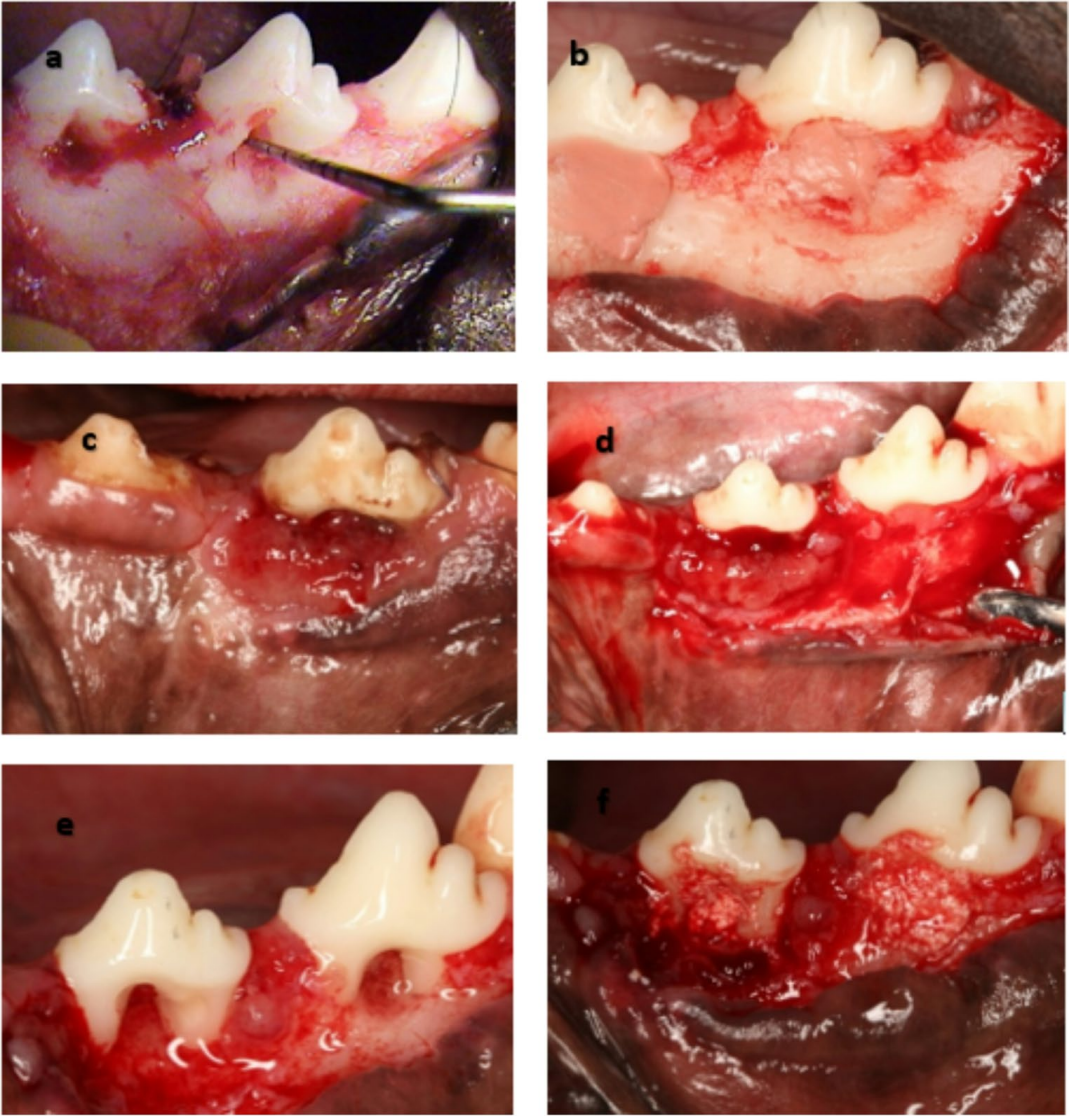
**a**: Creation of class II furcation buccal defects) At P2 and P3 roots with Periodontal prob confirm the horizontal defect depth (3 mm). **b**: A rubber base impression material was placed in the furcation defect to induce a chronic defect. **c**: Interrupted suture with complete coverage of the defect. **d**: After one month, a full-thickness flap was elevated, and the impression material was removed. **f**: the granulation tissue was removed, and the exposed root surfaces were scaled and planed. **e**: The furcation defects were filled by the β-tricalcium phosphate/gelatin composite scaffolds with or without Gentamycin according to the treatment option for each test group

Full-thickness flap surgery was performed to create 24 buccal Class II furcation defects in mandibular second and third premolars (P2 and P3) bilaterally. A standard defect measuring 5 mm in height, measured using a periodontal probe from the furcation fornix to the base of the defect, and 3 mm in depth, measured from the buccal surface, was created (Fig. 1a). To prevent the occurrence of spontaneous repair, a rubber base impression material was placed in the furcation area to induce an inflammatory effect and plaque accumulation for one month (Selvig 1994) [33] (Fig. 1b). The flaps were repositioned and sutured using a non-absorbable suture (5 − 0 Seralon, Germany) (Fig. 1c). All animals were fed a soft diet for two weeks to allow plaque accumulation and gingival inflammation.

After one month, a full-thickness flap was elevated, the impression material was removed, the granulation tissue was removed, and the exposed root surfaces were scaled and planed. (Fig. 1d and e) The furcation defects were randomly assigned to the test and control sides using the split-mouth design. The furcation defects of one group of teeth on the test sides were treated with β-tricalcium phosphate/gelatin composite scaffolds with Gentamycin (Test1 *n* = 8) embedded in the class II furcation defects (**Group I)**. (Fig. 1f) The furcation defects of the other test group were treated with β-tricalcium phosphate/gelatin composite scaffolds without Gentamycin (**Group II)**, being embedded in class II furcation defects. The control group received OFD only (**Group III)**. Ketoprofen for analgesia (Capisten IM 50 mg, 2 mg/kg, 0.1 mL/kg; Kissei Pharmaceutical) intramuscularly every 12 h for pain control and tetracycline hydrochloride 125 mg IM for the first two days (Shirakata et al., 2022) [34]. The antibiotic was mixed in the dogs’ food for seven days. Dogs were fed a soft diet in the postoperative period to reduce the possibility of local trauma to the operated sites [35].

### Histological preparations

The animals were sacrificed with an overdose of thiopental sodium after 8 weeks, and the surrounding bone and the tissues of the furcation defect were dissected and processed for histological evaluation. The blocks were promptly fixed for one week in 4% buffered formaldehyde. The specimens were next dehydrated in rising ethanol concentrations (50, 70, 90, and 100%) using a dehydration machine (ASP 300 S, Leica Biosystems) with agitation and vacuum. The blocks were embedded in clear chemically polymerized methyl methacrylate resin and cut into a coronal-apical plane using a precision-cutting machine (Metkon’s Micracut150 precision cutter), sections were then polished with 800-grit silicon carbide paper. After staining (Stevenel’s blue and Van Gieson picrofuchsin), the sections were examined using a light stereomicroscope (BX61; Olympus Corp) equipped with a high-resolution digital camera (E330; Olympus Corp).

### Histomorphometry analysis

**Fig. 2:**
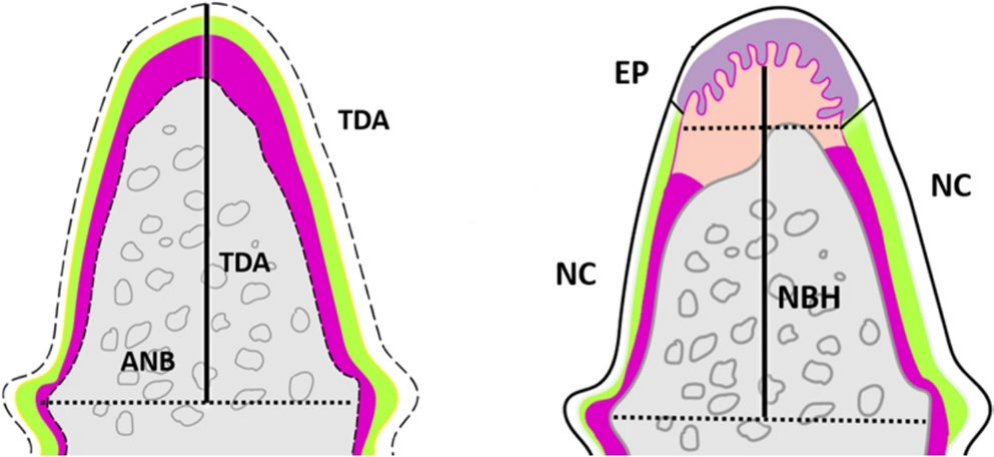
Schematic representation of the area and linear measurements evaluated in histometric analysis [Total defect area (TDA), Total defect height (TDH), Area of newly formed bone (ANB), Epithelial down growth (EP), New cementum (NC), Newly formed bone height (NBH)]

Histomorphometric analysis was done blindly using image analysis (Image Focus Alpha software, ImageFocus Alpha Software). The fornix of the furcation and root notches were used as reference points. Histometric analysis was conducted using three serial sections representing the central portion of the furcation site [35]. The evaluator was blinded to the treatment assigned and did not know the group that the histological section belonged to. The following measurements were made (Fig. 2):

1. Total area of the furcation defect (TDA; mm2) is the area measured from the apical line between the two notches and the root surface in the furcation region.
2. Percentage value of the area of the newly formed bone (NFBA): NFBA/TDA x 100.
3. Defect height (DH): the distance between the furcation fornix and the midpoint of a line connecting the notches (mm).
4. Percentage value of the newly formed bone height (NBH): NBH/DH x 100.
5. Percentage value of the newly formed cementum (NFAC): length of the NFAC/length of the root from the furcation fornix to the bottom of the notch x 100.
6. Percentage value of the newly formed periodontal tissues (NFPL) representing an area coronal to the notch: length of the newly formed PDL/length of the root from the fornix of the furcation to the bottom of the notch x 100.
7. Percentage value of the length of the epithelial down growth (EP): length of the EP in the defect/length of the root from the fornix of the furcation to the bottom of the notches x 100.

### Statistical analysis

Data were analyzed using the full detailed form: SPSS 24, IBM, Armonk, NY, United States of America. Quantitative data were expressed as mean ± standard deviation (SD), median, and interquartile range (IQR). The following tests were done:

1. Z: Mann-Whitney test for abnormally distributed quantitative variables, to compare between two groups. **Normality testing**: The **Shapiro–Wilk test** was conducted to assess data distribution. Results indicated that the data were not normally distributed (*P* <.05), which justified the use of non-parametric tests (Kruskal–Wallis and Mann–Whitney U). H: Kruskal-Wallis test for abnormally distributed quantitative variables, to compare between more than two studied groups.
2. **Dunn–Šidák correction**: this method was used to adjust the significance threshold for multiple pairwise comparisons following the Kruskal–Wallis test, thereby reducing the likelihood of Type I error.

## Results

**Fig. 3:**
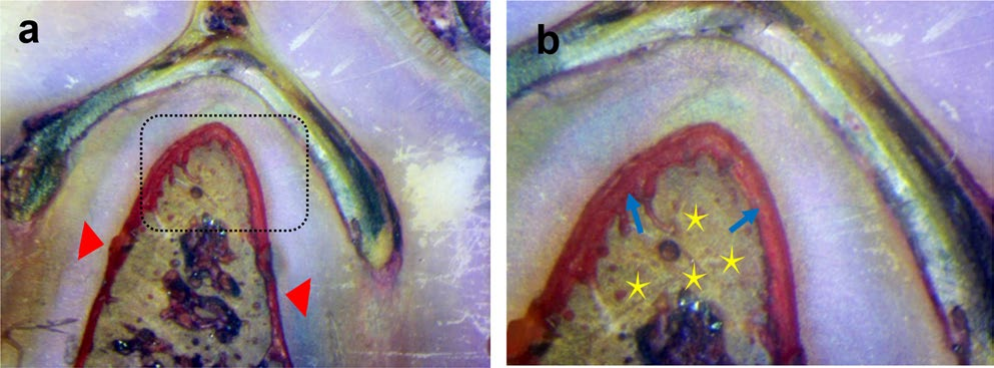
Undecalcified section of mandibular premolar teeth of the dog treated by β-tricalcium phosphate/gelatin composite scaffolds incorporated with gentamycin-loaded chitosan microspheres (Group I) after 8 weeks postoperatively revealed good healing with an extensive amount of newly formed bone (yellow asterisks) with inserted newly formed PDL fibers (blue arrows). The notches created on the root surface were covered by new cementum (red arrows). (a x200 & b x400)

**Fig. 4:**
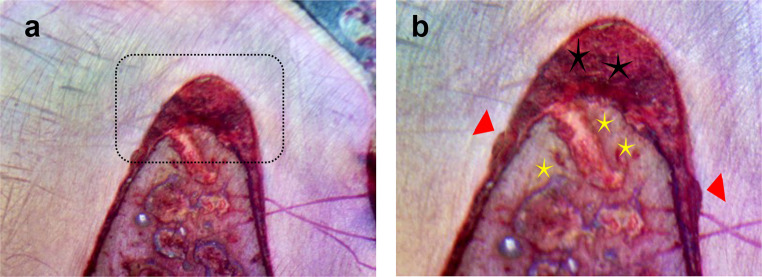
Undecalcified sections of mandibular premolar teeth of the dog treated by β-tricalcium phosphate/gelatin composite scaffolds without Gentamycin (Group II**)** after 8 weeks postoperatively showed a large area of granulation tissue (black asterisks) in the defect with partial bony restoration (yellow asterisks). The notches were made on the root surface (red arrows). (a x200 & b x400)

The healing process proceeded without any noticeable complications, such as infection or suppuration, with no exposure of the scaffolds during the healing period until all the animals were sacrificed. The histological findings of group I (β-tricalcium phosphate/gelatin composite scaffolds incorporated with gentamycin-loaded chitosan microspheres) revealed good healing with an extensive amount of newly formed bone with regenerated newly formed PDL fibers. The notches produced on the root surface were covered by new cementum there were collagen fiber bundles of variable thickness inserted into the new cementum. Figure 3-A and B. Group II (β-tricalcium phosphate/gelatin composite scaffolds without Gentamycin) showed the presence of a large area of granulation tissue in the defect with partial bony restoration, Fig. 4-C and D.

**Fig. 5:**
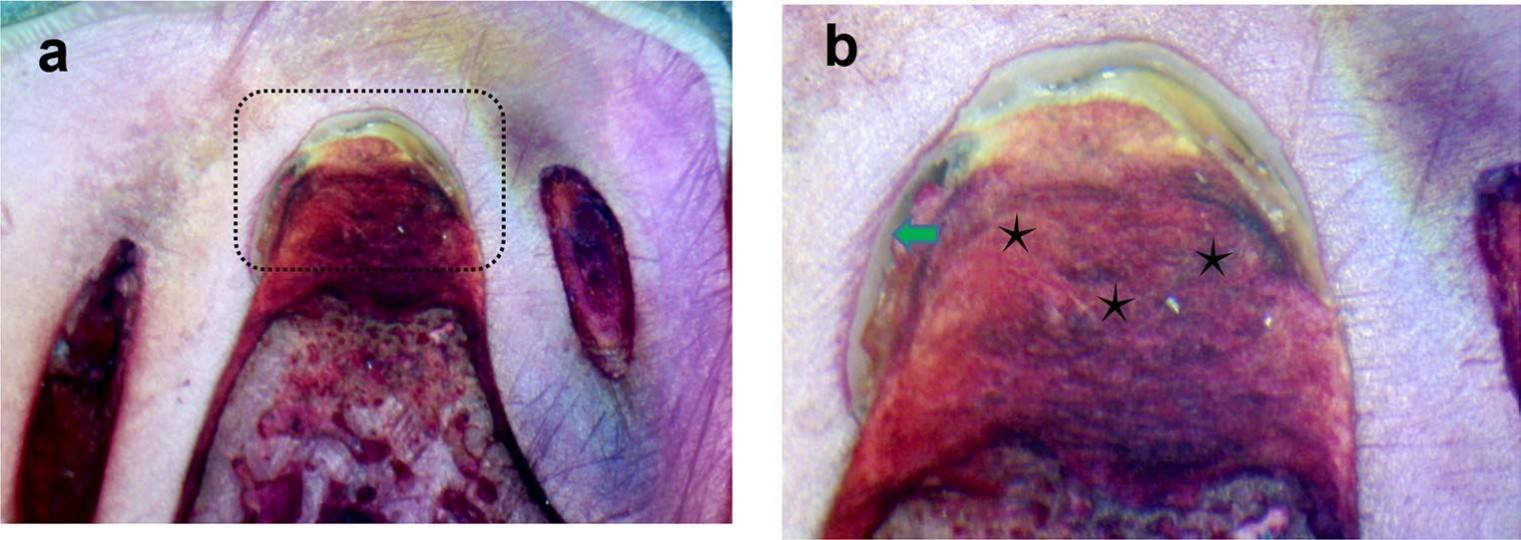
Undecalcified sections of mandibular premolar teeth of the dog treated by open flap debridement only (Group III) after 8 weeks postoperatively showed a great area of granulation tissues (black asterisks) and an area of epithelial down growth (green arrow). (a x200 & b x400)

**Fig. 6:**
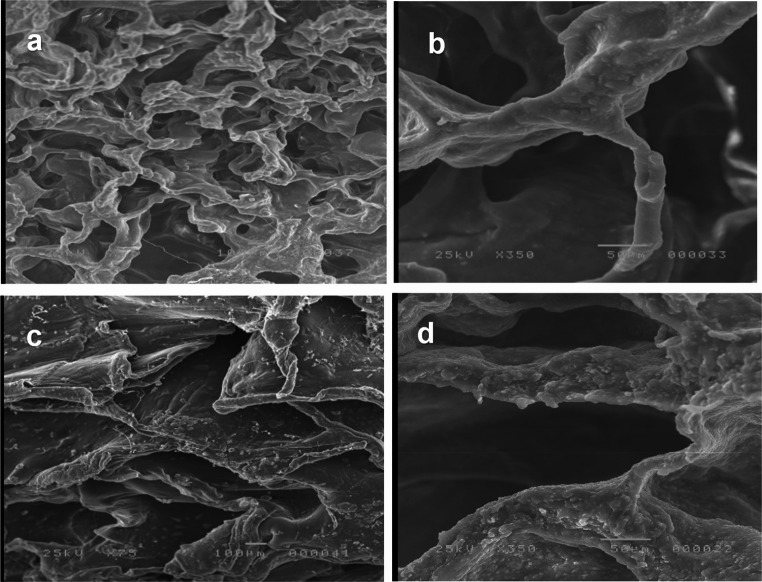
SEM images of CMs (GM)-β-TCP/gelatin composite scaffolds a(X75) &b(X350), c &d: CMs-β-TCP/gelatin composite scaffolds c (X 75) & d (X350)

On the other hand, the control group (open flap debridement) showed a very minimal amount of new bone, which was limited to the base of the defect, Fig. 5-A. There was no evidence of new bone regeneration in the defect center. In addition, the cavity was filled with fibrous connective tissue beside the great area of granulation tissues, and an area of epithelial down growth was also observed, Fig. 5-A and B. SEM images revealed that the scaffold’s internal structure consisted of a network of interconnected pores with uniformly sized openings with precipitation of calcium and phosphate ions on the surface of the scaffold, Fig. 6-A and D. There was a significant statistical difference in NBH (H(df = 2) = 13.564, *P* <.001) as the highest value was observed for group I (53.89 ± 5.02), while for Group II it was 36.39 ± 4.81, and for group III it was the lowest (23.01 ± 19.33). There was also a significant statistical difference in the percentage of the Newly Formed Bone area (H(df = 2) = 15.420, *P* <.001), as the highest value was observed for group I (40.87 ± 1.35), followed by group II (32.14 ± 3.06), whereas group III was associated with the lowest value (8.25 ± 12.63). Regarding the percentage value of the Newly Formed Cementum (%NFAC), there was no significant difference (H(df = 2) = 15.420, *P* <.001) between group I and II (39.49 ± 3.67), while group III was associated with the lowest values (17.16 ± 4.70). Group I also demonstrated the highest percentage value of the newly formed periodontal tissues (44.21 ± 2.56), followed by group II (34.63 ± 4.19) and finally the lowest values associated with group III (16.21 ± 5.93).

A different pattern was observed for the percentage value of the length of the epithelial down growth (%EDG), as group I was associated with the lowest value (125 ± 0.354), followed by group II (0.661 ± 0.563) and finally group III (21.146 ± 1.832). All previous data are summarized in Tables 1 and 2.

**Table 1:** Histomorphometric measurements (mean ± SD, median [IQR]) of newly formed bone height, bone area, cementum, and periodontal tissues in the three study groups at 8 weeks. Statistical analysis performed using Kruskal–Wallis test followed by Mann–Whitney U test with Dunn–Šidák correction for multiple comparisons. Normality was assessed using the Shapiro–Wilk test

		β-tricalcium phosphate/gelatin composite scaffolds incorporated with gentamycin- loaded chitosan microspheres (GI)	β-tricalcium phosphate/gelatin composite scaffolds loaded chitosan microspheres (GII)	Open flap debridement (GIII)	Test of significance *p* value
		*N* = 8	*N* = 8	*N* = 8	
percentage of the Newly Formed Bone Height (NBH)	**Min – Max** **Mean ± SD.** **Median** **95% CI for median** **25th Percentile** **– 75th Percentile**	46.87–59.45% 53.89 ± 5.02 56.14 47.36–58.04 48.68–57.59	30.23–43.24% 36.39 ± 4.81 35.47 31.70–41.66.70.66 32.52–40.83	0.00–59.45.00.45% 23.01 ± 19.33 23.03 0.00–33.33.00.33 8.33–30.95	H_(df=2)_ = 13.564 *p* =.001*
percentage of the Newly Formed Bone area (NFBA)	**Min – Max** **Mean ± SD.** **Median** **95% CI for median** **25th Percentile** **– 75th Percentile**	38.70–43.18.70.18% 40.87 ± 1.35 40.66 40.00–41.86.00.86 40.14–41.73	27.04–36.85% 32.14 ± 3.06 32.92 29.41–34.09 29.92–33.78	0.00–38.70.00.70% 8.25 ± 12.63 4.65 0.00–8.66.00.66 2.01–7.01	H_(df=2)_ = 15.420 *p* <.001*
percentage of the Newly Formed Cementum (NFAC)	**Min – Max** **Mean ± SD.** **Median** **95% CI for median** **25th Percentile** **– 75th Percentile**	33.40–44.79.40.79 39.49 ± 3.67 40.18 35.60–42.30 36.90–41.80	33.40–44.79.40.79 39.49 ± 3.67 40.18 35.60–42.30 36.90–41.80	10.00–22.72.00.72 17.16 ± 4.70 17.27 11.81–22.72 13.63–21.36	H_(df=2)_ = 15.420 *p* <.001*
percentage of the Newly Formed Periodontal Tissues (NFPL)	**Min – Max** **Mean ± SD.** **Median** **95% CI for median** **25th Percentile** **– 75th Percentile**	39.30–47.91.30.91 44.21 ± 2.56 44.80 43.80–47.91.80.91 43.00–45.45.00.45	26.04–39.58 34.63 ± 4.19 34.89 32.29–38.54 33.33–37.50	10.90–27.00 16.21 ± 5.93 13.65 11.81–23.63 12.27–19.99	H_(df=2)_ = 19.862 *p* <.001*
percentage of the length of the epithelial downgrowth (EP)	**Min – Max** **Mean ± SD.** **Median** **95% CI for median** **25th Percentile** **– 75th Percentile**	0–1 0.125 ± 0.354 0 −0.171-0.421 0–0	0–1.2 0.661 ± 0.563 0.895 0.191–1.132 0.0–1.175.0.175	18.75–23.95 21.146 ± 1.832 21.35 19.615–22.678 19.348–22.65	H_(df=2)_ = 18.129 *p* <.001*

n: Number of specimens

Min-Max: Minimum – Maximum

CI: Confidence interval

df = degree of freedom

H: Kruskal-Wallis Test

NS: Statistically not significant *(p* ≥.05)

*Statistically significant *(p* <.05)

**Table 2:** Pairwise intergroup comparisons (Mann–Whitney U test with Dunn–Šidák correction) of histomorphometric parameters among group I (β-TCP/gelatin + gentamycin-loaded Chitosan microspheres), group II (β-TCP/gelatin scaffolds without gentamycin), and group III (Control: open flap debridement). Data expressed as mean ± SD, median [IQR]

	GI vs. GII	GI vs. GIII	GII vs. GIII
Bone height (mm)	Z = 3.361 *p* =.001*	Z = 2.557 *p* =.010*	Z = 2.261 *p* =.021*
Bone area (mm^2^)	Z = 3.329 *p* =.001*	Z = 3.313 *p* <.001	Z = 2.522 *p* =.012*
Cementum (mm)	Z = 0.000 *p* = 1.00 NS	Z = 3.401 *p* =.002*	Z = 3.401 *p* =.002*
PL (mm)	Z = 3.260 *p* =.001*	Z = 3.363 *p* <.001*	Z = 3.258 *p* =.001*
epithelial down growth (mm)	Z = 1.707 *p* =.263 NS	Z = 4.268 *p* <.001*	Z = 2.561 *p* =.031*

NS: Statistically not significant *(p* ≥.05)

*Statistically significant *(p* <.05)

n: Number of specimens

Min-Max: Minimum – Maximum

CI: Confidence interval

df = degree of freedom

H: Kruskal-Wallis Test

NS: Statistically not significant *(p* ≥.05)

*Statistically significant *(p* <.05)

## Discussion

The ideal approach to treating infected bone defects should involve both controlling the infection and repairing the bone defect at the same time. Consequently, there is a pressing need for biomaterials that possess dual capabilities of bone regeneration and infection control. Carriers for local antibiotic delivery should fulfill several criteria: they must deliver antibiotics at an appropriate rate for treating infection, promote bone regeneration, and possess adequate mechanical strength to support physiological loads [36]. The present study demonstrates that β-tricalcium phosphate/gelatin composite scaffolds incorporating gentamicin-loaded chitosan microspheres promote enhanced periodontal regeneration in Class II furcation defects compared to scaffolds without gentamicin and open flap debridement. Regenerative outcomes—including bone formation, cementogenesis, and periodontal ligament regeneration—can be attributed to the synergistic properties of the scaffold components. β-TCP provides osteoconductivity and mechanical stability, gelatin serves as a biocompatible matrix supporting cell attachment and proliferation, and chitosan microspheres allow controlled local delivery of gentamicin, which contributes to infection control and supports the healing environment (Liu et al., 2022; Habib et al., 2021) [19, 32]. Prior studies have confirmed that gentamicin-loaded chitosan microspheres effectively reduce bacterial colonization in bone defects, highlighting the dual regenerative and antimicrobial function of this system (Thompson et al., 2019) [14].

The experimental Class II furcation model was designed to mimic the chronic inflammatory environment observed in human periodontal furcation defects. The placement of rubber base impression material in the furcation induced plaque accumulation and sustained inflammation, creating conditions that closely approximate clinical pathology (Selvig, 1994) [33]. This model allows evaluation of scaffold performance under conditions where regeneration is typically compromised, providing relevant translational insights.

The microsphere-based delivery system within the β-TCP/gelatin scaffold offers several advantages over conventional scaffolds. Mechanically, the scaffold maintains the defect space and supports cellular infiltration, while the microspheres enable sustained local release of gentamicin, minimizing systemic exposure and targeting bacterial infection directly at the defect site. The uniform pore network facilitates cell migration, vascularization, and direct osteogenesis, enhancing tissue integration and functional regeneration (Omata et al., 2014; Shujaa Addin et al., 2017) [37, 38]. These combined features make this approach particularly promising for chronic periodontal defects and peri-implant bone deficiencies, where infection and bone loss coexist.

The current study aimed to assess the potential effects of β-tricalcium phosphate/gelatin composite scaffolds incorporated with gentamycin-loaded chitosan microspheres, β-tricalcium phosphate/gelatin composite scaffolds incorporated with chitosan microspheres, and Open flap debridement on periodontal regeneration in critical-sized Class II furcation defects in an animal model. Undecalcified bone histology is a valuable diagnostic tool for the evaluation of the bone microstructure. It shows both the mineralized and cellular components of bone, which provide vital information on bone turnover or bone formation and resorption. It also assesses the interfaces between bone, implants, and the surrounding soft tissues. Additionally, it is a time-reducing method [7, 38–40]. The test sites treated with β-tricalcium phosphate/gelatin composite scaffolds incorporated with gentamycin-loaded chitosan microspheres exhibited statistically significantly greater amounts of newly regenerated bone height, new functional attachment cementum (NFAC), and the percentage value of the area of the newly formed bone (NFBA) and newly formed periodontal tissues (NFPL) compared to sites treated -tricalcium phosphate/gelatin composite scaffolds incorporated with chitosan microspheres and with open flap debridement (OFD).

However, there was no statistically significant difference between the β-tricalcium phosphate/gelatin composite scaffolds incorporated with gentamycin-loaded chitosan microspheres and β-tricalcium phosphate/gelatin composite scaffolds loaded with chitosan microspheres. This is explained by the fact that using scaffolds and MSs together is a powerful preparation that can quicken the osteoconductive and osteoinductive processes [39–43]. Additionally, TCP is a biomaterial with good osteoconductive and osteoinductive properties that can be degraded in vivo [7, 44]. On the other hand, the uniformly sized pores that are interconnected play a crucial role in tissue regeneration, as they are essential for cell migration and proliferation, ultimately leading to direct osteogenesis [19].

Epithelial down-growth was significantly lower in defects treated with β-TCP/gelatin scaffolds, with or without gentamicin-loaded chitosan microspheres, compared to the open flap debridement group. This indicates that the scaffolds effectively maintained the regenerative space, acting as a barrier to epithelial migration and preventing gingival connective tissue ingrowth, which is critical for predictable periodontal regeneration (Shue et al., 2012) [39]. Qualitative histological observations suggested minimal inflammatory cell infiltration in the scaffold-treated groups, whereas mild to moderate inflammation was observed in the control group. Although quantitative assessment of inflammatory markers was not performed, these findings support the scaffold’s role in providing a stable, infection-controlled environment conducive to tissue regeneration. Future studies should incorporate standardized scoring systems or immunohistochemical markers to objectively evaluate inflammation and its impact on regenerative outcomes. MSs fabricated using CS can also play an antibacterial role in the degradation process by locally controlled release of antibiotics. Results proved that CMs (GM)–β-TCP/gelatin composite scaffolds had contributed to the efficacious management of chronically infected class II furcation defects due to the infection control by the release of gentamycin. Chitosan microspheres loaded with β-tricalcium phosphate/gelatin composite scaffolds exhibited greater improvement than the control. The presence of infection in the control group and the antibacterial action of chitosan in β-tricalcium phosphate/gelatin composite scaffolds loaded with chitosan microspheres could be the cause of this. Liu et al. (2022) [19] created β-tricalcium phosphate/gelatin composite scaffolds that included CMs(GM)-β-TCP/gelatin microspheres loaded with gentamycin. The goal of these scaffolds was to maximize the release of gentamycin locally in areas with infected defects and to encourage bone regrowth.

The results of this study indicate that the innovative composite scaffold has the potential as a therapeutic option for the treatment of bone defects and abnormalities caused by infection. In a study conducted by Soundrapandian et al., [45] the bioactivity of the bioactive glass-based scaffold was examined by an in vitro acellular method. The scaffolds were loaded with two different drugs, an antibacterial or antifungal drug. The effects of the size of the scaffold, drug concentration, and dissolution medium on drug release were studied. The scaffolds were further coated with a degradable natural polymer, chitosan, to further control the drug release. The scaffolds released both the drugs for 6 weeks, in vitro. The results indicated that the bigger the size and the higher the drug concentration, the better the release profile. The scaffolds appeared to be suitable for local delivery of the drugs in cases of osteomyelitis, Omata et al., [37] confirmed that gelatin/β-TCP composites facilitate the controlled release of biologically active fibroblast growth factor (FGF). This controlled release mechanism is achieved through the biodegradation of the composites, ultimately promoting bone regeneration.

Furthermore, these findings align with those of Hoshi et al., [46] and Shujaa Addin et al., [38], who investigated the combined use of recombinant human FGF-2 and gelatin/β-TCP sponge in recession defects and ridge augmentation in dogs, respectively. Both studies demonstrated that the experimental sites exhibited enhanced tissue regeneration, characterized by significantly larger amounts of new cementum and new bone compared to control sites. Regarding epithelial down growth, the control group (OFD) demonstrated a statistically significantly higher amount of percentage value of the length of the epithelial down growth (EP) than the test groups. Scaffolds can prevent epithelial migration, stop the ingrowth of gingival connective tissue, and protect formed blood clots. Maintaining the regenerative space as a barrier against epithelial down-growth will provide predictable, satisfactory outcomes. A limitation of the present study is the absence of quantitative in vitro data on gentamicin release from scaffolds. Although the scaffolds incorporated gentamicin-loaded chitosan microspheres and demonstrated improved regenerative outcomes, the release kinetics, whether burst or sustained, were not measured. Future studies should evaluate the antibiotic release profile using standardized in vitro assays to better correlate drug release behavior with antimicrobial efficacy and scaffold functionality. Additionally, while qualitative observations of inflammatory cell infiltration were made during histological evaluation, no objective quantification of inflammation was performed. As the degree of inflammation can critically influence periodontal regeneration, future work should incorporate standardized histological scoring systems or immunohistochemical markers (e.g., CD45, IL-1β, TNF-α) to provide a more comprehensive assessment of the inflammatory response in both control and experimental groups. Addressing these points will strengthen the mechanistic understanding and clinical relevance of the scaffolds.

## Conclusions

Within the limitations of this study, β-TCP/gelatin scaffolds incorporating gentamicin-loaded chitosan microspheres provide a multifunctional platform that combines structural support, infection control, and promotion of periodontal tissue regeneration. The experimental outcomes indicate their potential as a clinically relevant approach for managing chronic furcation defects and other periodontally compromised sites. Future studies should focus on optimizing antibiotic release profiles, quantifying inflammatory responses, and evaluating long-term functional outcomes to support translation into human clinical applications.

## Supplementary Information

Below is the link to the electronic supplementary material.

Supplementary Material 1

## Data Availability

The data that support the findings of this study are available from the corresponding author upon reasonable request.

## References

[1] Kornman KS, Giannobile WV, Duff GW (2000) Quo vadis: what is the future of periodontics? How will we get there?. Periodontol 2017. 75(1):353–371 10.1111/prd.12217 28758298

[2] Nishikawa T et al (2012) Confocal laser scanning microscopy in study of bone calcification. Appl Surf Sci 262:64–68

[3] Annabi N et al (2010) Controlling the porosity and microarchitecture of hydrogels for tissue engineering. Tissue Engineering Part B: Reviews 16(4):371–383 20121414 10.1089/ten.teb.2009.0639 PMC2946907

[4] Chapple IL et al (2015) Primary prevention of periodontitis: managing gingivitis. J Clin Periodontol 42:S71–S76 25639826 10.1111/jcpe.12366

[5] Weiland AJ, Phillips TW, Randolph MA (1984) Bone grafts: a radiologic, histologic, and biomechanical model comparing autografts, allografts, and free vascularized bone grafts. Plast Reconstr Surg 74(3):368–379 6382367

[6] Sakai K (2011) Effects on bone regeneration when collagen model polypeptides are combined with various sizes of alpha-tricalcium phosphate particles. Dent Mater J 30(6):913–922 22123017 10.4012/dmj.2011-126

[7] Helal MH (2019) Osteogenesis ability of CAD-CAM biodegradable polylactic acid scaffolds for reconstruction of jaw defects. J Prosthet Dent 121(1):118–123 29961633 10.1016/j.prosdent.2018.03.033

[8] Hutmacher DW (2000) Scaffolds in tissue engineering bone and cartilage. Biomaterials 21(24):2529–2543 11071603 10.1016/s0142-9612(00)00121-6

[9] Albrektsson T et al (1981) Osseointegrated titanium implants. Requirements for ensuring a long-lasting, direct bone-to-implant anchorage in man. Acta Orthop Scand 52(2):155–170 7246093 10.3109/17453678108991776

[10] Wirth C (2008) Biomaterial surface properties modulate in vitro rat calvaria osteoblasts response: roughness and or chemistry? Materials Science and Engineering: C 28(5):990–1001

[11] Helal MH et al (2024) Prefabricated CAD-CAM scaffolds for management of oro-antral communication: a case report and histological analysis. Clin Implant Dent Relat Res 26(2):258–265 38225873 10.1111/cid.13300

[12] Alder KD (2020) Intracellular *Staphylococcus aureus* in bone and joint infections: a mechanism of disease recurrence, inflammation, and bone and cartilage destruction. Bone 141:115568 32745687 10.1016/j.bone.2020.115568

[13] Mohamed W (2014) Intracellular proliferation of *S. aureus* in osteoblasts and effects of rifampicin and gentamicin on *S. aureus* intracellular proliferation and survival. Eur Cells Mater 28:258–268 10.22203/ecm.v028a18 25340805

[14] Thompson K et al (2019) Intraoperative loading of calcium phosphate-coated implants with gentamicin prevents experimental *Staphylococcus aureus* infection *in vivo*. PLoS ONE 14(2):e0210402 30707699 10.1371/journal.pone.0210402 PMC6358082

[15] Ledesma F (2022) Nanomaterial strategies for delivery of therapeutic cargoes. Adv Funct Mater 32(4):2107174

[16] Su Y (2021) PLGA-based biodegradable microspheres in drug delivery: recent advances in research and application. Drug Deliv 28(1):1397–1418 34184949 10.1080/10717544.2021.1938756 PMC8248937

[17] Rizeq BR et al (2019) Synthesis, bioapplications, and toxicity evaluation of Chitosan-based nanoparticles. Int J Mol Sci. 10.3390/ijms20225776 31744157 10.3390/ijms20225776 PMC6888098

[18] Eivazzadeh-Keihan R et al (2022) Review: the latest advances in biomedical applications of chitosan hydrogel as a powerful natural structure with eye-catching biological properties. J Mater Sci 57(6):3855–3891

[19] Liu Y et al (2022) β-tricalcium phosphate/gelatin composite scaffolds incorporated with gentamycin-loaded chitosan microspheres for infected bone defect treatment. PLoS ONE 17(12):pe0277522 10.1371/journal.pone.0277522 PMC9731412 36480529

[20] Rezwan K (2006) Biodegradable and bioactive porous polymer/inorganic composite scaffolds for bone tissue engineering. Biomaterials 27(18):3413–3431 16504284 10.1016/j.biomaterials.2006.01.039

[21] Bohner M, Santoni BLG, Döbelin N (2020) β-tricalcium phosphate for bone substitution: synthesis and properties. Acta Biomater 113:23–41 32565369 10.1016/j.actbio.2020.06.022

[22] Geckil H (2010) Engineering hydrogels as extracellular matrix mimics. Nanomedicine 5(3):469–484 20394538 10.2217/nnm.10.12 PMC2892416

[23] Vaquette C et al (2012) A biphasic scaffold design combined with cell sheet technology for simultaneous regeneration of alveolar bone/periodontal ligament complex. Biomaterials 33(22):5560–5573 22575832 10.1016/j.biomaterials.2012.04.038

[24] Prabaharan M (2015) Chitosan-based nanoparticles for tumor-targeted drug delivery. Int J Biol Macromol 72:1313–1322 25450550 10.1016/j.ijbiomac.2014.10.052

[25] Dash M (2011) Chitosan—A versatile semi-synthetic polymer in biomedical applications. Prog Polym Sci 36(8):981–1014

[26] Bottino MC et al (2012) Recent advances in the development of GTR/GBR membranes for periodontal regeneration–a materials perspective. Dent Mater 28(7):703–721 22592164 10.1016/j.dental.2012.04.022

[27] Elgali I (2017) Guided bone regeneration: materials and biological mechanisms revisited. Eur J Oral Sci 125(5):315–337 28833567 10.1111/eos.12364 PMC5601292

[28] du Percie Sert N (2020) The ARRIVE guidelines 2.0: updated guidelines for reporting animal research. PLoS Biol 18(7):e3000410 32663219 10.1371/journal.pbio.3000410 PMC7360023

[29] Charan J, Biswas T (2013) How to calculate sample size for different study designs in medical research? Indian J Psychol Med 35(2):121–126 24049221 10.4103/0253-7176.116232 PMC3775042

[30] Tella EA et al (2023) Evaluation of hyaluronic acid gel with or without acellular dermal matrix allograft in the treatment of class II furcation defects in dogs: a histologic and histomorphometric study. Saudi Dent J 35(7):845–853 38025597 10.1016/j.sdentj.2023.07.007 PMC10658385

[31] Pannucci CJ, Wilkins EG (2010) Identifying and avoiding bias in research. Plast Reconstr Surg 126(2):619–625 20679844 10.1097/PRS.0b013e3181de24bc PMC2917255

[32] Habib S et al (2021) Antimicrobial activity, physical properties and sealing ability of epoxy resin-based sealer impregnated with green tea extract-chitosan microcapsules: an in-vitro study. Egypt Dent J 67(3):2309–2319

[33] Selvig KA (1994) Discussion: animal models in reconstructive therapy. J Periodontol 65(12):1169–1172 7877090 10.1902/jop.1994.65.12.1169

[34] Shirakata Y et al (2021) Periodontal wound healing/regeneration of two-wall intrabony defects following reconstructive surgery with cross-linked hyaluronic acid-gel with or without a collagen matrix: a preclinical study in dogs. Quintessence Int 52(4):308–316 33533237 10.3290/j.qi.b937003

[35] Fernandes JM et al (2005) Enamel matrix proteins associated with GTR and bioactive glass in the treatment of class III furcation in dogs. Braz Oral Res 19(3):169–175 16308603 10.1590/s1806-83242005000300003

[36] Chang W et al (2007) Adult osteomyelitis: debridement versus debridement plus osteoset T pellets. Acta Orthop Belg 73(2):238–243 17515238

[37] Omata K (2012) Enhanced bone regeneration by gelatin-β-tricalcium phosphate composites enabling controlled release of bFGF. J Tissue Eng Regen Med 8(8):604–611 22782937 10.1002/term.1553

[38] Shujaa Addin A et al (2017) Biodegradable gelatin/beta-tricalcium phosphate sponges incorporating recombinant human fibroblast growth factor-2 for treatment of recession-type defects: a split-mouth study in dogs. J Periodontal Res 52(5):863–871 28345758 10.1111/jre.12456

[39] Shue L, Yufeng Z, Mony U (2012) Biomaterials for periodontal regeneration: a review of ceramics and polymers. Biomatter 2(4):271–277 23507891 10.4161/biom.22948 PMC3568111

[40] Gao P et al (2011) Recent advances in materials for extended-release antibiotic delivery system. J Antibiot (Tokyo) 64(9):625–634 21811264 10.1038/ja.2011.58

[41] Gao P et al (2011) Recent advances in materials for extended-release antibiotic delivery system. J Antibiot 64(9):625–634 10.1038/ja.2011.58 21811264

[42] Kinoshita Y, Maeda H (2013) Recent developments of functional scaffolds for craniomaxillofacial bone tissue engineering applications. Sci World J 2013:p863157 10.1155/2013/863157 PMC3791836 24163634

[43] Chen KY (2009) Reconstruction of calvarial defect using a tricalcium phosphate-oligomeric proanthocyanidins cross-linked gelatin composite. Biomaterials 30(9):1682–1688 19136152 10.1016/j.biomaterials.2008.12.024

[44] Stevanović M et al (2018) Gentamicin-Loaded bioactive Hydroxyapatite/Chitosan composite coating electrodeposited on titanium. ACS Biomater Sci Eng 4(12):3994–4007 33418800 10.1021/acsbiomaterials.8b00859

[45] Soundrapandian C (2010) Porous bioactive glass scaffolds for local drug delivery in osteomyelitis: development and in vitro characterization. AAPS PharmSciTech 11(4):1675–1683 21107772 10.1208/s12249-010-9550-5 PMC3011056

[46] Hoshi S (2016) Ridge augmentation using recombinant human fibroblast growth factor-2 with biodegradable gelatin sponges incorporating β-tricalcium phosphate: a preclinical study in dogs. J Periodontal Res 51(1):77–85 26031712 10.1111/jre.12285

