# Supplementary figures and images for: β-tricalcium phosphate/gelatin composite scaffolds incorporated with gentamycin-loaded chitosan microspheres for periodontal regeneration in class II furcation defects in dogs
Source: Clin Oral Investig. 2025 Oct 29;29(11):540. doi: 10.1007/s00784-025-06582-0 (PMC12568831; doi:10.1007/s00784-025-06582-0)

**Ethical approval**

**
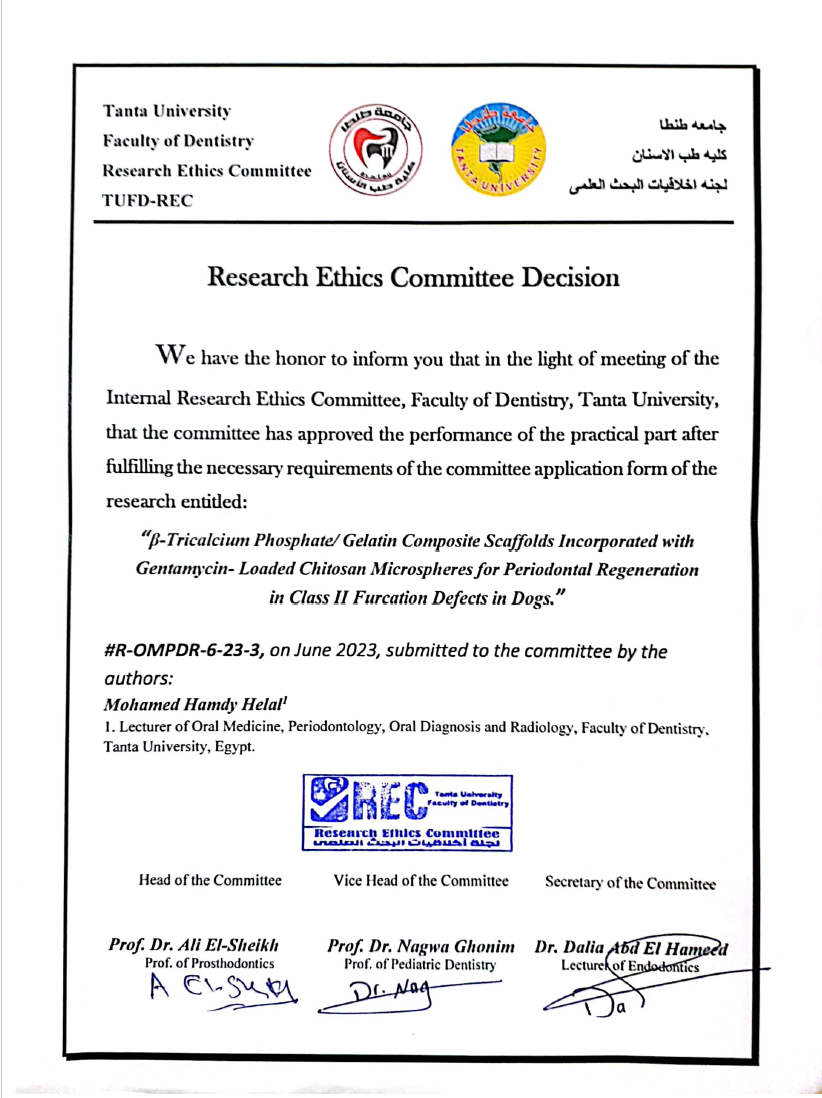
**

**Sample size calculation**


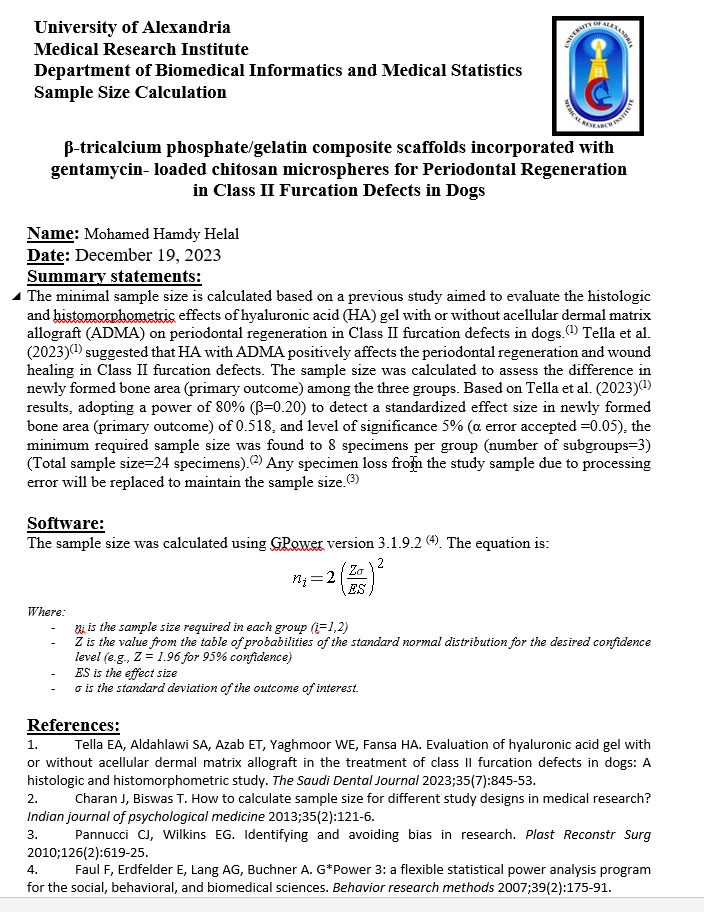


Arrive Guidelines Check List


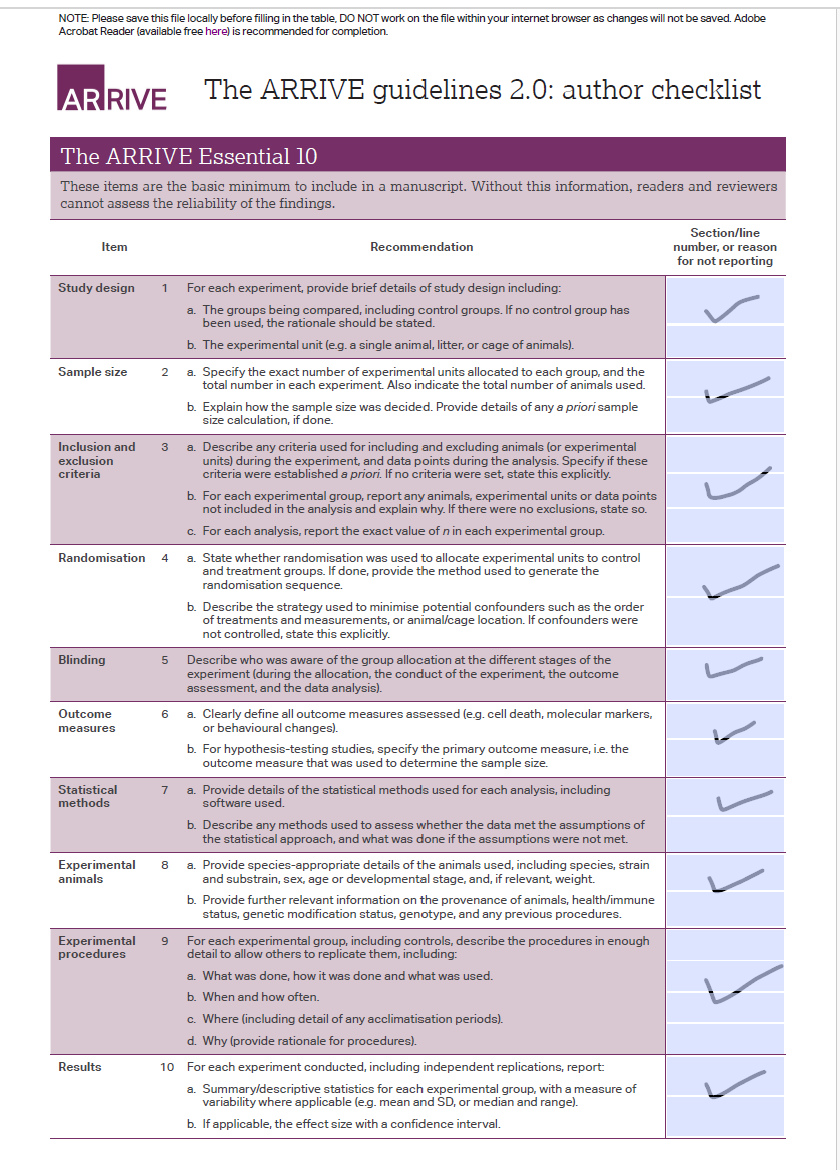

Supplement: Supplementary file 1 — Supplementary Material 1 [file 784_2025_6582_MOESM1_ESM.docx]
